# Can we monitor adaptation of juvenile goats to a new social environment through continuous qualitative behaviour assessment?

**DOI:** 10.1371/journal.pone.0200165

**Published:** 2018-07-06

**Authors:** Fabio Napolitano, Maria Serrapica, Ada Braghieri, Salvatore Claps, Francesco Serrapica, Giuseppe De Rosa

**Affiliations:** 1 Scuola di Scienze Agrarie, Forestali, Alimentari ed Ambientali, Università degli Studi della Basilicata, Potenza, Italy; 2 CREA Research Centre for Animal Production and Acquaculture, Bella Muro, Potenza, Italy; 3 Dipartimento di Agraria, Università degli Studi di Napoli Federico II, Portici, Naples, Italy; University of Illinois, UNITED STATES

## Abstract

We aimed to verify whether Continuous Qualitative Behaviour Assessment (10 observers used a list of six qualitative descriptors) paired with Temporal Dominant Behavioural Expression (the same observers were asked to select the dominant descriptor and to score its intensity level) was able to monitor fluctuations of animal behaviour expression over time. We applied these techniques to three groups of juvenile goats either weaned (group C), or un-weaned (groups WOM and WM). Each animal was separated from its group, moved to group C and tested for 30 min either while their mothers were at pasture, or while their mothers were in an adjacent pen (group WOM and WM, respectively). Animals from group C were separated from their group and immediately reintroduced to it. TDBE duration and score of each descriptor of behavioural expression were able to detect differences among groups but were unable to describe how the behaviour of the goats changed as the time progressed. TDBE curves described the evolution of each behavioural expression of each animal over time but were unable to detect differences among groups. The **χ**^2^ test conducted on peaks of dominance, albeit displaying the variations of the behavioural expression over time and allowing the assessment of differences among groups, focussed on occurrences of higher agreement between observers while neglecting most of the information concerning the descriptors above the level of significance. Conversely, based on mixed analysis of variance with the fixed effects of group, test interval and group x test interval (animal nested into group and observer were considered to be random), most of the descriptors were able to discriminate the three experimental groups while preserving the information on the fluctuations of the behavioural expression of the animals during the test.

## Introduction

Goats are social and gregarious animals [1] and a stable social environment may promote their adaptation to the environment, through social learning, and their welfare conditions, as suggested by reduced responsiveness to stressing events. Conversely, social disturbance, such as the inclusion of a new subject in a stable social group, can cause increased aggression in most farm animal species including goats with potential negative effects on animal welfare and production [2]. These effects are attributed to the alteration of the social structure of the group and increased aggression levels remain visible as long as a new social hierarchy is established. According to Alley and Fordham [3], the introduction of an unknown animal in a group of feral goats causes increased aggressiveness; however, social disturbance cannot be detected after 24 h. Similarly to sheep [4], goats are considered socially tolerant animals [5]. However, there is no information about how and when aggressive interactions cease after the alteration of the social hierarchy of a group of domestic goats.

Qualitative Behaviour Assessment (QBA) is able to describe the way an animal, or a group of animals, interacts with the environment: the method focusses on how the animals express their behaviour rather than on what they do [6]. This method proved to be valid and reliable in a number of farm and companion animal species, including goats [7], and was successfully used to assess the effect of various environmental challenges, such as isolation [8], handling [9], transport [10]. In addition, Rousing and Wemelsfelder [11] applied this method to describe the social behaviour of dairy cattle and found significant correlations with quantitative behavioural data. QBA in its original construction is able to perform a post hoc evaluation of animal behaviour and observers are asked to summarise the information gathered throughout the period of observation giving one score for each behavioural descriptor. Although QBA has been used in research for more than a decade [12], little is known on the potential of QBA for monitoring the fluctuations of the style of interaction of the animals with the environment. In food sensory science an innovative technique has been recently proposed in order to describe the development of the sensory experience while a food is consumed [13]. In a previous work, Napolitano et al. [14] combined QBA and TDS and proposed a continuous qualitative behaviour assessment (C-QBA) approach for the description of changes in animal behavioural expression. These authors observed that C-QBA was able to detect short-term variations in the behaviour of dairy buffaloes exposed to a novel environment. However, often social stabilisation, and the related changes in behavioural expressions, occurs in a time span, which is well beyond the limits imposed by feasibility on continuous recording. In addition, the statistics applied in that study did not allow a contemporary assessment of the effects of treatment and time. Therefore, we aimed to verify whether the method was able to monitor fluctuations of animal behaviour expression in a longer period of time while allowing a comparison of the evolution of the behavioural expression in different treatment groups.

## Material and methods

The Institutional Ethical Committee of Università della Basilicata approved this research.

### Experimental procedure

Thirty female juvenile “Rossa Mediterranea” goats aged 106.7 ± 2.1 days (mean ± SD) were provided by CREA (Research Centre for Animal Production and Acquaculture, Italy). Animals were balanced for age and weight and equally divided in three groups as follows: Control (C), Without-mother (WOM) and With-mother (WM). The three groups were housed in three adjacent straw-bedded pens (3 x 6 m for each pen) with open metal fencing. The pen of group C was located between the pens of groups WOM and WM. Juvenile goats abruptly weaned and separated from their mothers two weeks before the start of the experiment composed group C, whereas un-weaned animals constituted groups WOM and WM. During the night, the juveniles of these two latter groups were kept with their mothers. These mothers were alternately allowed to the pasture either during the morning or during the afternoon. Before testing two juvenile goats (1 from group WM and 1 from group WOM) were excluded from the trial due to health problems.

The tests were conducted in four 5-h morning sessions and testing order was randomized across groups. Each animal from groups WOM and WM was separated from its group, moved to group C and left there for 30 min. However, animals from group WOM were tested while the mothers were at pasture, whereas animals from group WM were tested while the mothers were in the home pen and could receive visual, olfactory and auditory signals from them. Animals from group C were separated from their group and immediately reintroduced to it. A red collar worn by each animal while tested made it identifiable. The same person performed all of the testing procedures; he was not involved in other farm and experimental practices.

All tests were video-recorded using a DVL-157 JVC video camera located at 3 m from the floor, equipped with a wide-angle lens and operated by remote control. The footage consisted of a 300, 270 and 270 min video for groups C, WM and WOM, respectively. From this material, 28 clips (ten animals in group C and 9 animals in groups WM and WOM) of 90 s each were created using the software AVS Video Converter. In particular, from each 30-min video three 30 s sequences were taken at 5, 15 and 25 min and joined to obtain one 90 s clip for each animal. Therefore, the total duration of the video material observed by the panel was 42 min (90 x 28 /60).

The study was conducted in compliance with the European legislation on the animals used both for scientific (Dir. 2010/63/UE) and farming purposes (Dir. 98/58/EC). In any cases, the experiment only involved procedures concerning handling and regrouping, which are comparable to those routinely used in commercial farms.

### Continuous qualitative assessment of behaviour

A ten-member panel (3 females and 7 males, aged 20 to 34 years) was used for continuous qualitative behaviour assessment (C-QBA). Panellists were graduate and undergraduate animal science students recruited from the University of Basilicata. Panellists were selected on the basis of their willingness to participate, experience in the observation of animal behaviour, ability in eliciting descriptors for qualitative behaviour assessment and capacity in discriminating animal responses to different environmental conditions using a predetermined list of descriptors.

Panellists were trained in eight sessions of 45 min each. The first session was dedicated to the choice of the descriptors to be used during C-QBA. The observers watched 20 sequences of 15 s representative of the behaviour of the animals during the tests but not included in the clips to be qualitatively assessed. At the end of each sequence, the panel briefly discussed and agreed about the terms better describing the behaviour previously observed. Six behavioural descriptors were chosen according to the frequency of elicitation performed by the panel during the training. They were: “calm”, “curious”, “aggressive”, “passive”, “alert” and “sociable”. The definitions of behavioural descriptors are reported in Table 1. Such definitions were used for the subsequent assessor training and were also available during the experimental observations.

**Table 1:** Descriptors and definitions used for assessor training to continuous qualitative behaviour assessment.

Descriptor	Definition
**Calm**	The animal is laying, standing or moving slowly with head raised. No overt signs of vigilance and/or agitation are visible
**Curious**	The animal is explorative, walking or standing and sniffing with lowered head various environmental stimuli including the fence and the ground.
**Aggressive**	The animal is aggressive and initiates agonistic encounters with co-specifics
**Passive**	The animal is submissive and subjected to agonistic interactions from co-specifics
**Alert**	The animal stands still with elevated neck and intently oriented head
**Sociable**	The animal is sociable and initiates or receives socio-positive contacts

The second and third sessions were used to train the panellists on the use of the Temporal Dominant Behavioural Expression (TDBE) system of data acquisition. Five additional sessions were used to teach the assessors how to continuously evaluate the behavioural descriptors to be used in the C-QBA. In session four (learning phase) the assessors watched six clips of 10–15 s corresponding to the standard for each behavioural descriptor (i.e. descriptor expressed at high intensity). The aim of this session was the memorisation of the six behavioural descriptors based on the observation of the clips of the standards with the definition of the corresponding descriptors. During sessions five and six the assessors observed 12 clips of 10–15 s corresponding to the behavioural descriptors at medium and high intensity. The observers assessed each single clip and indicated the dominant descriptor with the help of the definitions. In the last 2 sessions, the observers assessed the same clips and were asked to identify the dominant behavioural descriptor and score its perceived intensity.

After the training, the clips were evaluated following the TDBE procedure, as described by Napolitano et al. [14]. The panellists watched each clip while the entire list of behavioural descriptors was available on the same computer screen. Assessors were then asked to select the dominant descriptor and to score its intensity level on a 100-mm unstructured scale anchored at the two extremities (0 mm: descriptor absent, 100 mm: descriptor very strong). A qualitative behavioural descriptor was scored as dominant when it attained most of the attention of the assessor (i.e. the most relevant impression at each given time). Subsequently, whenever deeming that the behavioural expression changed, either in quality or in intensity, the assessor scored the new dominant descriptor and/or level, until the end of the clip. Descriptors could be chosen several times within each clip, while other descriptors could be entirely neglected. Each clip was observed in quadruplicate (i.e. 4 times by each observer) in a randomised order. The order of the descriptors did not change within each observer in order to simplify the scoring procedure but it was randomised across assessors. Each assessor observed twelve clips in each session. Five min intervals were given between consecutive series of four clips. A total of twenty-eight clips by four replications were observed in 10 sessions of about 50 min each in order to stay above the threshold of 30 evaluations for each descriptor and each animal (10 observers x 4 replications = 40 evaluations), as suggested by Pineau et al. [15] for sensory attributes.

### Continuous quantitative assessment of behaviour

The behaviour expressed by the experimental animals in the 30-min clips (n = 28) was quantitatively analysed through continuous recording using the software The Observer XT version 8.0 (Noldus Information Technology, The Netherlands). All the observations were performed by the same trained observer. Training consisted in the observation of 6 clips (two for each group) with the aim to instruct the observer in recognizing the behavioural categories identified in previous ad libitum sampling sessions concerning all the recorded material. The recorded mutually exclusive behaviours are briefly described in Table 2. The mutually exclusive postures of standing and laying were also recorded (laying was excluded from the analysis because it represents the complement of standing and it doesn’t provide any further information). Both behaviours and posture are indicated in the text as quantitative variables.

**Table 2:** Definitions of behavioural categories continuously recorded using The Observer XT (Noldus Information Technology, The Netherlands).

Behavioural category	Description
**Inactive, min**	No overt activity, the animal is standing or lying,
**Walking, min**	The animal walks slowly, looking around or in front
**Exploration, min**	The animal walks slowly with the neck horizontal often stopping and sniffing the ground or the fence
**Eating (min)**	Standing still while selecting and ingesting feed
**Socio-positive behaviour, min**	Sniffing or nuzzling conspecifics
**Agonistic behaviour, min**	Pushing, butting or threatening conspecifics
**Vigilance, min**	Standing still with elevated neck and intently oriented head
**Orientation toward home pen, min**	Standing with head, including eyes and ears, oriented toward the home pen
**Vocalization, n**	Emission of acoustic signals
**Flight attempts, n**	The animal runs towards the fence but suddenly stops before colliding

### Statistical analyses

TDBE data were gathered and analysed using the software FIZZ (Biosystemes, Couternon, France), which allowed computing the total dominance duration in s for each descriptor, observer, clip and replication. The proportion of runs (observer x replication) in which each descriptor was selected (i.e. perceived as dominant by the observer) was recorded at 2 s intervals and indicated as dominance rate (%). As a result, for each animal the TDBE curves were constructed. In addition, chance level and significance level were computed and reported. Chance level is the dominance rate that can be obtained by chance taking into account the number of descriptors included in the test. In this study chance level was 0.167 (P_0_ = 1/p, where p is the number of descriptors), corresponding to a dominance rate of 17%. Descriptors with dominant rates below the chance level can be considered negligible. Significance level is calculated as follows:

$${\text{P}}_{s}={\text{P}}_{0}+1.645\surd \frac{P_{0}\left(1-P_{0}\right)}{n},$$

where n = number of observers × replications. It represents the minimum value of dominance rate to be reached by a descriptor to be considered significantly higher than the chance level. Therefore, TDS curves above the significance level are consistent across the panel [13–14]. In this study the significance level was 0.268, corresponding to a dominance rate of 27%. At 2 s intervals the intensity level for each descriptor, observer, clip and replication was also recorded and, based on duration of dominance and intensity levels, the scores were calculated [16]. Dominance durations and scores of the descriptors are indicated in the text as qualitative variables.

The subsequent analyses were conducted using SAS software [17]. In order to verify panel performance across observers and replications, dominance rates and scores of each descriptor were subjected to a three-way analysis of variance using observer (10 levels), replication (4 levels), group (3 levels) and their first order interactions as factors (S1 Data).

The mean values of dominance rates and scores along with variables gathered through Observer XT (S1 Data) were subjected to one-way analysis of variance with group as factor (3 levels). When a significant effect of group was observed the Bonferroni t-test was used to locate differences between means. For the entire panel no animals of group C showed any aggressive behaviour. In addition, the quantitative variables flight attempts and vocalization were never detected in group C while in the same group Orientation toward the home pen was not recordable. Therefore, for the analyses of these variables we used only two levels (WM and WOM) for the factor group. In any cases, with this approach, the responses of observers were summarised throughout the acquisition time period and no time effect was evaluated.

Therefore, for each descriptor within each group, as suggested by Napolitano et al. [14], the changes of behavioural expressions during the test, based on the frequency of occurrences of peaks of dominance rates, were assessed using a 2 (peak of dominance presence vs. absence) x 3 (test intervals) χ^2^ test (S2 Data). Conversely, Dinnella et al.’s [18] suggestion was to identify relevant time periods, summarise dominance responses within these time periods and express them as frequency values for further analyses. In this study 3 time intervals were identified (0–30, 30.5–60, 60.5–90 s) as initial, mid and final phases of each test. Frequency values were then subject to a mixed procedure to assess the fixed effect of group (3 levels), test intervals (3 levels) and group x test interval. Animal nested into group (28 levels) and observer (10 levels) were considered to be random. Animal nested into group was utilised as the error term to test the effect of group (S3 Data). Also in this case, the variable aggressive behaviour was analysed using only two levels (WM and WOM) of the factor group. Least squares means estimates are reported. Significance and tendency were declared at *P* ≤ 0.05 and *P* ≤ 0.10, respectively.

## Results and discussion

### Reliability of the observers

No significant interaction group x replication was detected for the qualitative variables duration and score; therefore, the behaviour expressed by the animals from each group was not assessed differently in different replications. Although the interaction group x observer and observer x replication were often significant, the F of the factor group was at least 10 times higher than that of the interactions for all of the descriptors apart from sociable (Table 3), thus suggesting that the observer training was effective and allowed to achieve a satisfactory level of panel reliability for at least 5 out of six descriptors. For the descriptor sociable the F of the interaction group x observer is similar to the F of the factor group. This result could be attributed either to ineffective training or to a reduced influence of the effect group on this descriptor.

**Table 3:** F values of the descriptors used in the continuous qualitative assessment.

Variables	Group	Group x observer	Group x replication	Observer x replication
***Duration***				
**Calm**	134.75	0.67	0.22	0.17
**Curious**	21.55	1.63	0.29	0.66
**Aggressive**	280.86	1.15	0.89	0.72
**Sociable**	7.37	2.46	0.43	1.00
**Passive**	59.21	1.99	0.54	0.38
**Alert**	406.12	1.14	0.59	0.80
***Score***				
**Calm**	79.41	2.38	0.31	2.26
**Curious**	15.38	1.49	0.55	1.02
**Aggressive**	272.18	4.06	0.57	0.61
**Sociable Sociable s**	6.87	5.53	0.63	1.82
**Passive**	56.23	2.13	0.67	0.21
**Alert**	536.41	1.85	0.61	1.19

**Fig 1:**
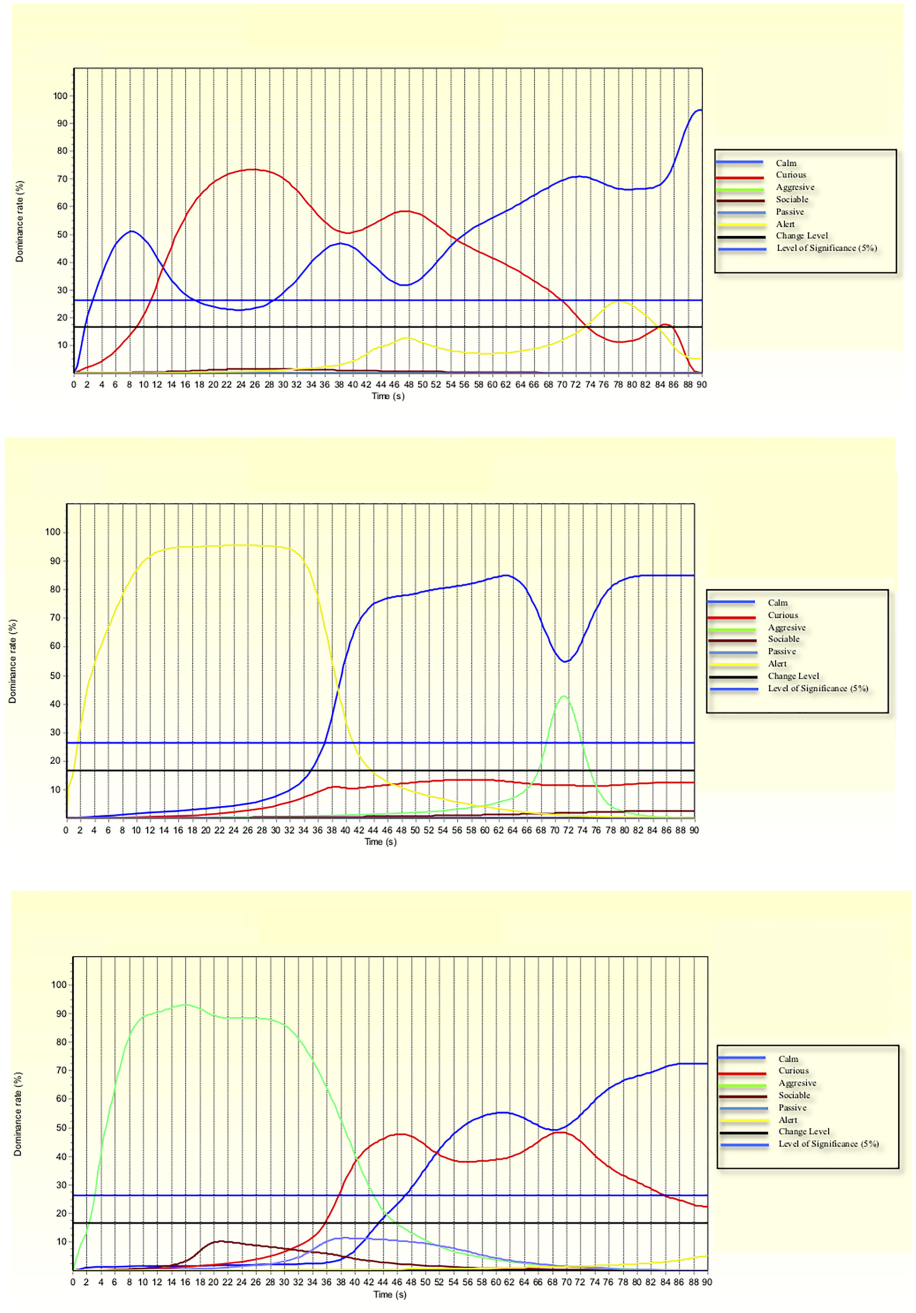
Temporal Dominant Behavioural Expression curves based on six descriptors showing the behavioural expressions of the animals 1 (a), 2 (b) and 3 (c) from the three treatment groups (C = Control, WOM = moved to the control group in absence of the mother, WM = moved to the control group in presence of the mother, respectively). The curves show the agreement among observers on the dominant descriptors as the test proceeded. When the curves are above the level of significance, descriptors are consistent across observers.

Observer reliability was also measured through the calculation of the dominant descriptors. In particular, the descriptors above the significance level (Fig 1) can be considered sufficiently consistent across the observers [13].

### Qualitative assessment of goat behavioural expression

Table 4 shows duration and score of the qualitative variables and the results of the one-way analysis of variance. The effect of group was significant for the descriptors calm (duration and score, P<0.01) and alert (duration and score, P<0.001), whereas the duration of the descriptor passive tended to be affected by group (P<0.10). Significant differences were observed between group C and group WM for the descriptor calm in terms of duration and score (P<0.05 and P<0.01, respectively), with higher levels in group C, whereas group C tended to show a higher level of the descriptor calm in terms of duration as compared with group WOM (P<0.10). No differences were observed between groups WOM and WM for the duration and score of the descriptor calm. These results indicate that, although separated from their group and then reintroduced to it, goats from group C managed to remain calm, whereas animals from the other two experimental groups, showed lower level of this descriptor as a consequence of their inclusion in a novel social environment. The duration and score of the descriptor alert were higher in group WM than in groups C and WOM (P<0.001), whereas no differences were observed between groups C and WOM. The different behaviour observed in group WM can be attributed to the fact that these un-weaned animals could receive visual, auditory and olfactory stimuli from their mothers and expressed their willingness to re-join them by attentively orienting towards the source of these stimuli. The analysis conducted on only two groups (WM and WOM) showed that the descriptor aggressive tended to be affected by group only in terms of duration (P<0.10). In particular, goats from group WOM tended to show higher levels of the descriptor aggressive as compared with group WM (P<0.10). The statistical analysis of the descriptor aggressive did not include Group C as these animals were never considered aggressive by the panel. The aggressiveness observed in groups WM and WOM perfectly matches the high level of the descriptor calm observed in group C and suggests that the reduced calmness in these two groups can be attributed to the agonistic behaviour expressed by the animals when temporary moved to an unfamiliar group (Group C) and forced to interact with unknown peers. The duration and score of the descriptor passive tended to be higher in group WOM than in group C (P<0.10), whereas no significant differences were observed between groups WM and C, and between groups WM and WOM. This result may be due to the fact that the lack of maternal stimuli induced the animals from group WOM to be less self-confident and more submissive when interacting with unknown co-specifics. No significant effect of the group was observed for the descriptors curious and sociable (duration and score).

**Table 4:** Mean ± SE of qualitative descriptors continuously recorded through Temporal Dominant Behavioural Expression (duration and score) and quantitative variables continuously recorded through The Observer XT of juvenile goats either reintroduced into their group (group C) or introduced into group C while the mothers were at pasture (group WOM) or introduced into group C while the mothers were in the home pen (group WM).

	C	WM	WOM	F	P
***Duration*, *s***					
**Calm**	48.75 ± 5.18	22.28 ± 5.46	31.51 ± 5.46	6.44	0.006
**Curious**	21.34 ± 3.79	13.26 ± 3.99	16.85 ± 3.99	1.09	0.352
**Alert**	9.53 ± 3.62	34.00 ± 3.82	4.22 ± 3.82	17.44	0.001
**Aggressive**	0.0	2.83 ± 2.90	10.66 ± 2.90	3.65	0.074
**Passive**	1.78 ± 3.16	6.62 ± 3.33	12.30 ± 3.33	2.63	0.092
**Sociable**	1.25 ± 0.79	2.31 ± 0.83)	3.70 ± 0.83	2.32	0.119
***Score***					
**Calm**	63.98 ± 4.56	40.49 ± 4.81	52.96± 4.81	6.27	0.006
**Curious**	35.15 ± 4.96	26.52± 5.23	27.51 ± 5.23	1.87	0.430
**Alert**	13.88 ± 3.92	53.27 ± 4.13	8.61 ± 4.13	35.32	0.001
**Aggressive**	0.0	7.06 ± 5.22	17.63 ± 5.22	2.05	0.171
**Passive**	4.44 ± 5.31	13.41± 5.60	22.20 ± 5.60	2.66	0.090
**Sociable**	4.57 ± 2.13	6.70 ± 2.24	10.03 ± 2.24	1.58	0.226
***Quantitative***					
**Standing, min**	22.88 ± 2.66	26.67 ± 2.80	27.42 ± 2.80	0.81	0.457
**Inactive, min**	14.60 ± 2.10	8.22 ± 2.21	7.75 ± 2.21	3.21	0.057
**Walking, min**	2.08 ± 0.51	1.67 ± 0.54	3.22 ± 0.54	2.24	0.127
**Exploration, min**	6.0.6 ± 0.93	2.48 ± 0.98	5.11 ± 0.98	3.73	0.038
**Eating, min**	6.33 ± 2.32	11.52 ± 2.44	8.87 ± 2.44	1.19	0.321
**Socio-positive behaviour, min**	0.55 ± 0.33	0.45 ± 0.35	1.49 ± 0.35	2.79	0.080
**Agonistic behaviour, min**	0.36 ± 0.42	0.91 ± 0.44	3.42 ± 0.44	13.99	0.001
**Vigilance, min**	0.01 ± 0.39	2.88 ± 0.41	0.08 ± 0.41	15.94	0.001
**Flight attempts, n**	0.0	0.44 ± 2.60	7.78 ± 2.60	3.98	0.063
**Vocalization, n**	0.0	37.66 ± 7.56	0.67 ± 7.56	11.97	0.003
**Orientation toward the home pen, min**	Not applicable	1.88 ± 0.59	0.05 ± 0.59	4.85	0.043

The results so far shown, displayed several significant differences between groups, but were unable to describe whether and how the behaviour of the goats changed as the time progressed. Fig 1(a), 1(b) and 1(c) displays the TDBE curves of three goats from groups C, WOM and WM, respectively. When descriptors presented dominance rates below 17% they were not considered dominant, whereas when showing dominance rates below 27% they were not considered significant. Fig 1a indicates that when re-introduced to the home pen this animal from group C was either calm (3 to 13 s and 55 to 90 s = 45 s in total) or curious (13 to 55 s = 42 s in total), while none of the other descriptors reached the significance threshold. The highest dominance rates were reached at 26 (73%) and 90 s (95%) for curious and calm, respectively. The juvenile goat from group WOM was introduced to a novel social environment while the mother was at the pasture (Fig 1b) and showed a different behavioural pattern: the first dominant descriptor (aggressive) was related to the agonistic interactions consequent to the introduction into a new social environment. This behavioural expression was dominant from 3 to 40 s with the highest rate at 16 s (93%); then curious prevailed (from 40 to 52 s) showing the highest dominance rate at 47 s (48%). The last dominant descriptor was calm with an increasing trend from 54 to 90 s when it reached a dominant rate of 73%. Fig 1c shows the behavioural expressions of an animal from group WM. Only two descriptors were dominant throughout the test. The first one (alert) was related to the separation of the un-weaned juvenile goats from their mothers while they could receive various maternal stimuli. This descriptor was dominant from 2 to 39 s with the highest dominance value of over 90% reached at 12 s and maintained up to 34 s; the second one (calm) was dominant from 40 to 90 s and reached the highest rate at 63 s with a value of 80%. This descriptor was over 70% for most of this period, although at around 72 s it decreased to 55% due to a corresponding increment of the descriptor aggressive, which became significant albeit not dominant with a maximum value of 43%. The curves of each animal are available but for sake of brevity those of the remaining 25 animals are not shown.

**Fig 2:**
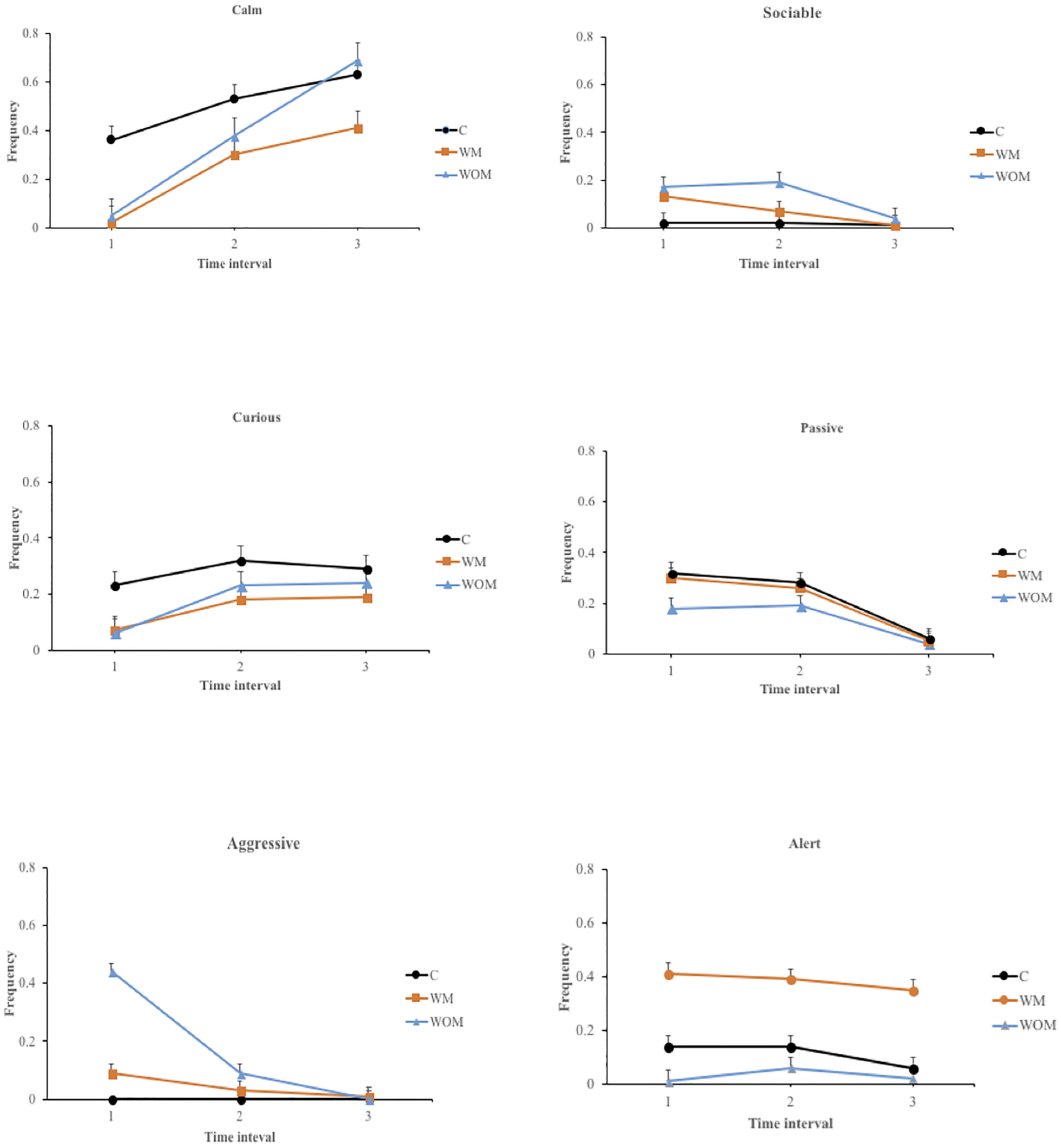
Temporal Dominant Behavioural Expression based on six descriptors and expressed by the three treatment groups (C = Control, WOM = moved to the control group in absence of the mother, WM = moved to the control group in presence of the mother, respectively) in three time intervals (1 = 0–30 s, 2 = 30.5–60 s, 3 = 60.5–90 s).

In order to statistically assess the effect of time, hence the adaptation of the goats to a novel social environment, we conducted a mixed analysis of variance as described in the section on statistical analyses and reported in Fig 2.

Based on this analysis, the descriptor calm showed significant effects of group (F_2,25_ = 4.95; P<0.05), time interval (F_2,797_ = 241.28; P<0.001) and their interaction (F_4,797_ = 16.01; P<0.001). In particular, group C showed higher levels of this descriptor as compared with WM (P<0.01), whereas no differences were observed between groups C and WOM as well as between WM and WOM. The level of the descriptor calm increased from the first to the second and the second to the third time interval (P<0.001). The significant interaction group x time interval can be attributed to the fact that at the first time interval the descriptor calm was higher in group C than in groups WM and WOM (P<0.01), whereas no significant differences between groups WM and WOM were observed. At the second and third time interval no differences were observed among the three groups. These results confirmed those obtained using one-way analysis of variance and added the information that the animals from group WOM and WM were able to reach the same levels of calmness as goats from group C by at the second time interval.

The descriptor curious was not affected by group (F_2,25_ = 1.97, NS) and the interaction time interval x group (F_4,797_ = 1.77; NS) but significantly affected by the time interval (F_2,797_ = 28.70; P<0.001). The descriptor curious was lower at the first time interval than at the second and third time intervals (P<0.001), whereas no differences were observed between the second and third time interval. Our results showed that animals became increasingly curious and explored more (see Table 1 for the definition of the descriptor curious) when they started to adapt to the testing conditions, i.e. when the level of neophobia decreased [19], which occurred at the second and third time interval.

The descriptor alert showed significant effects of group (F_2,95_ = 19.84; P<0.001) and time interval (F_2,797_ = 4.68; P<0.01), whereas the interaction was not significant (F_4,797_ = 1.71; NS). This descriptor was lower in groups C and WOM than in group WM (P<0.001), whereas no differences were observed between groups C and WOM. In addition, it was higher at the time intervals 1 and 2 than at time interval 3 (P<0.05 and P<0.01, respectively), whereas no differences were observed between time intervals 1 and 2. These results indicate that animals from group WOM were able to keep their behavioural expression of alertness at levels similar to those expressed by group C, whereas animals from group WM were induced by the perception of maternal stimuli to maintain a higher level of this behavioural expression throughout the test.

The descriptor aggressive showed significant effects of group (F_1,16_ = 7.85; P<0.01), time interval (F_2,509_ = 197.04; P<0.001) and their interaction (F_2,509_ = 92.86; P<0.001). In particular, in group WM the descriptor aggressive was lower than in group WOM (P<0.01). This descriptor was higher at the first time interval than at the second and third time interval and higher at the second than at the third time interval (P<0.001). We observed that the descriptor aggressive was lower in group WM than in group WOM (P<0.001) at the first time interval. This descriptor at the second and third time intervals showed no differences between groups. Again in un-weaned juvenile goats (i.e. groups WOM and WM) we observed a gradual transition from a conflictual situation, at the inclusion in the unfamiliar group (group C), to a more stable condition in the second time interval for goats from group WM and in the third time interval for goats from group WOM. Conversely, the temporary separation and subsequent reintroduction of individual goats from group C did not change the level of the descriptor aggressive, since the panel never described as aggressive these animals.

The descriptor passive showed significant effects of time interval (F_2,797_ = 31.65; P<0.001) and the interaction group x time interval (F_4,797_ = 10.00; P<0.001), whereas it tended to be affected by group (F_2,25_ = 2.66; P<0.10). Passive tended to be higher in group WOM than in group C (P<0.10). This descriptor was higher at the first time interval than at third time interval and higher at the second than at the third time interval (P<0.001), with no differences between first and second time intervals. No differences were observed in group C when the three time intervals were compared, whereas the descriptor passive decreased from first to the third time interval in group WM (P<0.001) and decreased from the first to the third and from the second to the third time interval in group WOM (P<0.001). The trend of this descriptor is similar that observed for the descriptor aggressive as the submissive behavioural expression more often observed soon after the inclusion of un-weaned juvenile goats into the control group changed to a more stable situation in the subsequent time intervals for groups WM and WOM, whereas animals from group C did not change this behavioural expression throughout the test.

The descriptor sociable showed significant effects of time interval and the interaction group x time interval (F_2,797_ = 106.31 and F_4,797_ = 11.42, respectively; P<0.001), whereas group was not significant (F_2,25_ = 2.22; NS). It was lower at time interval 1 than at time intervals 2 and 3 (P<0.001), whereas no significant difference was detected between time intervals 2 and 3. At the first time interval the descriptor sociable was lower in group WOM than in groups C and WM (P<0.05 and P<0.01, respectively), whereas no other significant differences were observed in this and the following time intervals. This result can be interpreted in terms of gradual integration within group C although this integration was slower for animals from group WOM, which were unable to receive any stimuli from their mothers. Unlike in previous studies on QBA conducted either using a fixed list [20] or based on free choice profiling [21], our approach allowed the description of the changes in behavioural expression over time while also differentiating the animals according to the treatment. As a consequence, QBA may represent a tool to provide relevant information about animal welfare even when changes in behavioural expression occur in response to changing environmental conditions [14].

The **χ**^2^ test on the frequency of occurrences of behavioural peaks showed that the dominance peaks of the descriptor calm had no changes throughout the test in group C (**χ**^2^ = 0.37, P = 0.83), whereas it increased from 11 to 56 and 78% in group WM (**χ**^2^ = 8.31, P = 0.02) and from 22 to 78 and 89% in group WOM (**χ**^2^ = 9.85, P = 0.01). In group WM the dominance of the descriptor curious tended to increase (**χ**^2^ = 5.01, P = 0.08) from 0 to 33 and 44, whereas no significant changes were observed in the groups C and WOM (**χ**^2^ = 1.20, P = 0.55 and **χ**^2^ = 2.49, P = 0.29, respectively). No significant changes were observed for the descriptor alert in all of the experimental groups, but for different reasons: a high percentage of peaks was observed in group WM (67% in all test intervals), and low percentages in groups C (20, 20, 10, **χ**^2^ = 0.48, P = 0.79) and WOM (0% in all test intervals). The descriptor aggressive showed no peaks in group C, decreased, albeit not significantly, in group WM (**χ**^2^ = 2.34, P = 0.30) and decreased in group WOM from 77 to 44 and 0 (**χ**^2^ = 11.35, P = 0.01). No significant changes were observed for the descriptor passive in groups WM and WOM (from 22 to 22 to 0, **χ**^2^ = 2.35, P = 0.31 and from 33 to 33 to 11, **χ**^2^ = 1.54, P = 0.46), whereas no peaks of the descriptor passive were detected in group C. The descriptor sociable never peaked in all of the experimental groups. The **χ**^2^ test on the frequency of occurrences of behavioural peaks yielded results similar to those previously described and obtained through mixed analysis of variance, although no comparisons among groups could be performed. In addition, this test focuses on occurrences of higher agreement between observers while neglecting most of the information available concerning the descriptors above the level of significance.

### Quantitative assessment of goat behaviour

We recorded quantitative variables (Table 2) throughout the duration of the test (30 min). Results are summarised in Table 4. The effect of group was significant for exploration (P<0.05), agonistic behaviour (P<0.001), vigilance (P<0.001), vocalisation (P<0.01) and orientation toward the home pen (P<0.05), whereas inactive and flight attempts tended to be affected by the group (P<0.10). Animals from Group C explored for longer than goats from Group WM (P<0.05). Agonistic behaviour was expressed for longer in animals from group WOM than in goats from Groups WM (P<0.01) and C (P<0.001). Vigilance lasted more in goats from Group WM than in goats from Groups C and WOM (P<0.001). Animals from Group WM vocalised more than animals from Group WOM (P<0.01), whereas goats from Group C did not vocalise at all. Goats from Group WM spent more time oriented towards the home pen than the goats from Group WOM (P<0.05). Animals from group C tended to be inactive for longer as compared with group WOM (P<0.10). The number of flight attempts tended to be higher in Group WOM than in Group WM (P<0.10), whereas no attempts were observed in Group C. Taken together these results appear to be in line with those gathered through C-QBA as showing a lower level of arousal in goats from group C, while groups WM and WOM were more involved in vigilance and agonistic behaviours, respectively.

## Conclusion

We conclude that the mixed analysis of variance of the descriptors evaluated using the C-QBA approach allowed a precise evaluation of the temporal evolution of the behavioural expression in different treatment groups. In addition, the identification of relevant time periods to be used for the analysis allowed to summarise the behavioural responses expressed by the animals over a relatively long period of time (30 min). Therefore, C-QBA may represent a tool to provide additional information about animal welfare even when changes in behavioural expression occur in response to changing environmental conditions.

Importantly, different analyses (i.e. analysis of variance: no significant interaction group x replication, low significance of the interaction group x observer; calculation of the dominance rate: TDBE curves above the significance level) consistently showed that the panel used for qualitative assessment was reliable and suggest that the panel training was effective. However, TDBE duration and score of the qualitative variables were able to detect differences among groups but were unable to describe how the behaviour of the goats changed as the time progressed, whereas TDBE curves described the evolution of each behavioural expression over time but were unable to detect differences among groups and the **χ**^2^ test, albeit displaying the variations of the behavioural expression over time and allowing the assessment of differences among groups, focussed on occurrences of higher agreement between observers while neglecting most of the information concerning the descriptors above the level of significance. Conversely, based on mixed analysis of variance, most of the qualitative descriptors were able to discriminate the three experimental groups while preserving the information on the fluctuations of the behavioural expression of the animals during the test.

## Supporting information

S1 Data File used for the analysis of variance: Sheets 1 and 2 were used to perform one-way analysis of variance (with group as factor) of quantitative variables (gathered through Observer XT); sheets 3 and 4 were used to conduct a three-way analysis of variance of qualitative variables (with observer, replication, group and their first order interactions as factors) to verify panel performance. (XLSX)

S2 Data Frequency of occurrences of peaks of dominance rates subject to a 2 (peak of dominance presence vs. absence) x 3 (test intervals) χ^2^ test. (XLSX)

S3 Data Frequency values of qualitative variables subject to a mixed procedure to assess the fixed effect of group, test interval and group x test interval. Animal nested into group and observer were considered to be random.
(XLSX)
